# The evolutionary history of *Shigella flexneri* serotype 6 in Asia

**DOI:** 10.1099/mgen.0.000736

**Published:** 2021-12-14

**Authors:** Si-Nguyen T. Mai, Ladaporn Bodhidatta, Paul Turner, Sonam Wangchuk, Tuyen Ha Thanh, Phat Voong Vinh, Duy Thanh Pham, Maia A. Rabaa, Guy E. Thwaites, Nicholas R. Thomson, Stephen Baker, Hao Chung The

**Affiliations:** ^1^​ Oxford University Clinical Research Unit, Ho Chi Minh City, Vietnam; ^2^​ Groningen Institute for Evolutionary Life Sciences, University of Groningen, Groningen, The Netherlands; ^3^​ Armed Forces Research Institute of Medical Sciences, Bangkok, Thailand; ^4^​ Cambodia-Oxford Medical Research Unit, Angkor Hospital for Children, Siem Reap, Cambodia; ^5^​ Centre for Tropical Medicine and Global Health, Nuffield Department of Clinical Medicine, University of Oxford, Oxford, UK; ^6^​ Royal Centre for Disease Control, Ministry of Health, Thimphu, Bhutan; ^7^​ The Wellcome Trust Sanger Institute, Hinxton, Cambridge, UK; ^8^​ London School of Hygiene and Tropical Medicine, Bloomsbury, London WC1E 7HT, UK; ^9^​ Department of Medicine, Cambridge Institute of Therapeutic Immunology and Infectious Diseases (CITIID), University of Cambridge, Cambridge, UK

**Keywords:** antimicrobial resistance, reductive evolution, *Shigella *genomic, *Shigella *phylogeny, *Shigella flexneri *serotype 6, *Shigella *evolution

## Abstract

*
Shigella flexneri* serotype 6 is an understudied cause of diarrhoeal diseases in developing countries, and has been proposed as one of the major targets for vaccine development against shigellosis. Despite being named as *S. flexneri *, *Shigella flexneri* serotype 6 is phylogenetically distinct from other *S. flexneri* serotypes and more closely related to *S. boydii *. This unique phylogenetic relationship and its low sampling frequency have hampered genomic research on this pathogen. Herein, by utilizing whole genome sequencing (WGS) and analyses of *Shigella flexneri* serotype 6 collected from epidemiological studies (1987–2013) in four Asian countries, we revealed its population structure and evolutionary history in the region. Phylogenetic analyses supported the delineation of Asian *Shigella flexneri* serotype 6 into two phylogenetic groups (PG-1 and −2). Notably, temporal phylogenetic approaches showed that extant Asian *S. flexneri* serotype 6 could be traced back to an inferred common ancestor arising in the 18^th^ century. The dominant lineage PG-1 likely emerged in the 1970s, which coincided with the times to most recent common ancestors (tMRCAs) inferred from other major Southeast Asian *S. flexneri* serotypes. Similar to other *S. flexneri* serotypes in the same period in Asia, genomic analyses showed that resistance to first-generation antimicrobials was widespread, while resistance to more recent first-line antimicrobials was rare. These data also showed a number of gene inactivation and gene loss events, particularly on genes related to metabolism and synthesis of cellular appendages, emphasizing the continuing role of reductive evolution in the adaptation of the pathogen to an intracellular lifestyle. Together, our findings reveal insights into the genomic evolution of the understudied *Shigella flexneri* serotype 6, providing a new piece in the puzzle of *Shigella* epidemiology and evolution.

## Data Summary

Raw sequence data used in this publication are available in the NCBI Sequence Read Archive (project PRJEB5281: Phylogeography of *Shigella spp*. in Southeast Asia and PRJEB2508: Temporal and geographical of the *Shigella* genus diversity in Southern Vietnam). All supporting data and protocols have been provided within the article or through supplementary data files.

**Impact Statement**

The bacterial genus *Shigella* inflicts a great burden of diarrhoeal diseases globally, particularly on young children in developing countries. Proposed pan-* Shigella* vaccine design should cover the four most common serotypes, namely *S. sonnei *, *S. flexneri* 2a, *S. flexeri* 3a, and *S. flexneri* serotype 6. While detailed genomic studies have unravelled the life histories of the former three serotypes, not much is known about the diversity and evolution of *S. flexneri* serotype 6. Herein, we used whole genome sequencing data of *S. flexeri* serotype 6 collected in four Asian countries to study its evolution in the region. We showed that the pathogen’s dominant lineage likely emerged in the 1970s, coinciding with the emergence timeline estimated previously for other major *S. flexneri* serotypes in Southeast Asia. This indicates the intensity of multiple *Shigella* introduction events into Asia post 1970s. Genomic analyses predicted that resistance against first-line antimicrobials (used for shigellosis treatment) was rare among the studied *S. flexneri* serotype 6. Our findings represent a novel understanding on the evolution of an understudied *Shigella* serotype, and provided an initial framework for future genomic epidemiology studies on this elusive pathogen.

## Introduction

*Shigella *, a member of the Gram-negative Enterobacteriaceae, is among the leading aetiologies responsible for diarrhoeal diseases. It has been estimated that shigellosis accounts for >160000 deaths annually worldwide, among which one-third were children under 5-years-old residing in developing countries [1, 2]. The *Shigella* genus is categorized into four species (or subgroups) based on the lipopolysaccharide O-antigen composition on the bacterial cell surface, including: *S. dysenteriae* (subgroup A), *S. flexneri* (subgroup B), *S. boydii* (subgroup C), and *S. sonnei* (subgroup D). Each subgroup is further classified into various serotypes according to type-specific antigens. The numbers of serotypes also vary between different subgroups (* S. boydii *: 20 serotypes, *S. dysenteriae *: 15 serotypes, *S. flexneri *: 14 serotypes, and *S. sonnei *: one serotype).

Despite a comprehensive serotyping scheme, it has been demonstrated that serotyping is a poor predictor of phylogenetic relatedness for *S. flexneri *, regardless of whether it is phenotypically or *in silico* determined [3]. Although *S. flexneri* serotype 6 (Sf6) is serologically classified as a *S. flexneri* serotype [4–7], multiple phylogenetic studies (based on multi-locus sequence typing (MLST) and whole genome sequencing (WGS) data) have demonstrated that Sf6 is distantly related to all other *S. flexneri* serotypes, and instead has closest phylogenetic relatedness to *S. boydii* [8–11]. Such distinct phylogenetic relationships with other *Shigella* species, however, have hampered research on Sf6. Species-wide studies on *S. flexneri* genomic and evolution, at both regional and global scale, have excluded Sf6 from in-depth analyses [12, 13]. On the other hand, research on *S. boydii* is currently limited due to its much lower contribution to disease burden over the past decade, compared to *S. flexneri* and *S. sonnei* [14, 15]⁠. However, unlike *S. boydii *, Sf6 is prevalent in shigellosis-endemic regions in Africa and Asia, accounting for ~11 % of documented shigellosis cases in a large scale surveillance study [15, 16]. Thus, Sf6 was proposed as one of the four essential targets (in conjunction with *S. sonnei *, *S. flexneri* 2a, and *S. flexneri* 3a) for developing a quadrivalent vaccine with broad coverage against diverse *Shigella* serotypes [15].

In spite of its epidemiological significance, there have been no large scale genomic studies focusing on the evolutionary history of Sf6, especially in Asia – a contemporary focus of endemic shigellosis. Herein, we performed WGS on a collection of representative Sf6 (*n*=96) isolated in South and Southeast Asia (collected between 1987 and 2013), and explored their phylogenetic structure and evolutionary history in the region. We also inspected their genetic repertoires and antimicrobial resistance profiles (resistomes). Finally, to further contextualize Sf6 evolution, our results were discussed in relation to previous findings in other *Shigella* species, as well as other enteric pathogens.

## Methods

### Bacterial isolates and whole genome sequencing

In order to investigate the evolutionary history of Sf6 in Asia, we compiled a collection of 96 Sf6 isolates (Table S1, available in the online version of this article) from four countries: Thailand, Vietnam, Cambodia, and Bhutan. The isolates originated from multiple collaborative institutes, including: Armed Forces Research Institute of Medical Sciences (AFRIMS) in Bangkok, Thailand (*n*=53); Oxford University Clinical Research Unit (OUCRU) in Ho Chi Minh City, Vietnam (*n*=30); Cambodia-Oxford Medical Research Unit (COMRU) in Siem Reap, Cambodia (*n*=6); Jigme Dorji Wangchuk National Referral Hospital (JDWNRH) in Thimphu, Bhutan (*n*=7). Serotyping was performed on all retrieved isolates at the collaborating institutes using the commercial antisera (Denka Seiken, Japan), and confirmed that they belong to Sf6. The bacterial isolates and data used in this study originated from several local diarrhoeal surveillance studies, which received ethical approvals from the Hospital for Tropical Diseases in Ho Chi Minh City, Vietnam, all other participating hospitals, the Institutional Review Board of the Walter Reed Army Institute of Research (for Thailand data), the Research Ethics Board of Health in Bhutan, and the Oxford Tropical Research Ethics Committee (OxTREC) in the United Kingdom. The study also included characterization of bacterial isolates submitted for routine diagnostic activities (for Cambodian data). All examined Sf6 were isolated from patients with diarrhoea, predominantly young children, during the diarrhoea surveillance studies. Written informed consent from study participants or their parents/guardians was obtained prior to the collection of stool samples.

We extracted genomic DNA for all bacterial isolates using Wizard Genomic DNA extraction kits (Promega, Wisconsin, USA), and DNA was stored at −20 °C until shipment to the Wellcome Trust Sanger Institute for whole genome sequencing on an Illumina HiSeq2000 platform. This produced a library of paired-end reads of 125 bp in length for each bacterial isolate.

### Short read mapping and phylogenetic reconstruction

The chromosome sequence of *S. boydii* serotype 4 Sb227 (accession number: CP000036.1) was used as the reference genome for mapping of all Sf6 since it was the most closely related complete genome at the time of analysis [9, 11]. The BWA-MEM algorithm (v0.7.12) [17] was used for short read mapping. Duplicate reads were removed by PICARD (v2.18.5) (https://github.com/broadinstitute/picard), and GATK (v3.7) [18] was deployed for indel realignment. Using SAMtools and bcftools (v1.8) [19], high-quality SNPs were called and filtered. Unqualified single nucleotide polymorphism (SNP) sites were discarded if they matched any of the following criteria: consensus quality <50, mapping quality <30, ratio of SNPs to reads <75%, and read depth <4.

Following the read mapping process, we created an alignment of pseudogenome sequences of the same length. For phylogenetic inference, recombination and prophage regions were removed from the alignment, resulting in an alignment of 3794 recombination-free bp. Prophage regions were detected on the reference chromosome sequence using the PHASTER web server [20], and recombination elements were predicted by Gubbins (v1.4.5) [21]. The best-scoring maximum-likelihood phylogenetic tree was inferred using RAxML (v8.2.4) under the GTRGAMMA substitution model, with 100 rapid bootstrap searches and 20 maximum-likelihood searches [22]. The resulting phylogeny was comprised of two clusters, separated by significant genetic distance. We herein refer to them as the PG-1 (*n*=82) and PG-2 (*n*=14). The maximum-likelihood phylogenetic tree was midpoint rooted in FigTree (v1.4.3) (http://tree.bio.ed.ac.uk/software/figtree/) for the purpose of visualization. Metadata were annotated to the phylogenetic tree and visualized using the ggtree package (v1.14.6) [23] in the R programming platform [24].

### Temporal structure analysis and Bayesian phylogenetic inference

In order to analyse potential temporal structure in the phylogeny, we subsampled the Sf6 collection to include 82 representative sequences. In the subsampling process, we included one representative isolate (and excluded the other(s)) from each terminal phylogenetic groups with bootstrap support less than 70. The intention of this subsampling procedure was to yield a phylogeny with high confidence support for the temporal structure analysis and subsequent Bayesian phylogenetic inference. A maximum-likelihood phylogeny was reconstructed for this subsampled dataset, using the approach described above. The most appropriate substitution model was determined by the ModelFinder algorithm of IQTree (v1.6.7) [25]. TempEst (v1.5.3) [26] was utilized to assess the linear relationship between root-to-tip divergence of the inferred maximum-likelihood phylogeny and year-of-sampling, which yielded a signal of high temporal structure (R^2^=0.864). We deployed BEAST (v1.8.3) [27] to co-infer the phylogeny and divergence times, which estimated the substitution rate and time to the most recent common ancestor (tMRCA) by Bayesian phylogenetic inference. To select the best-fit model, analyses were performed independently on six combinations of different models of molecular clock (strict, uncorrelated relaxed, and random local clock) and demographic models (constant model and Bayesian Skyline). Analyses on each model combination were performed in triplicate on an ensemble of 400 million continuous Markov Chain Monte Carlo (MCMC) chains, with samples taken every 40 000 chain generations. Convergence of estimated parameters were visually inspected in Tracer (v1.7.1) [28], ensuring the effective sample size (ESS) values of estimated parameters were ≥200 for a successful run. Among the tested models, parameters estimated on the combination of a random local clock and Bayesian Skyline did not successfully converge in three separate runs. Thereby, it was not considered in downstream analyses. We applied path sampling and stepping-stone sampling approaches in every BEAST run to approximate the marginal likelihood for model selection [29, 30], in order to identify the best-fit model. Parameters inferred from triplicate runs of the best-fit model were combined using LogCombiner, with 10 % burn-in removal, and a Maximum Clade Credibility tree was generated from the combined estimates using TreeAnnotator. The model incorporating the GTR + Γ4 substitution model, random local clock, and constant population size was chosen as best fitting to the data.

In addition, Bayesian inference was performed separately for PG-1 and PG-2 isolates, following the procedure as described above but without subsampling. For PG-1 analyses, two sequences were discarded in the input alignment since they showed high proportions of gaps and ambiguous nucleotides. BEAST runs on PG-1 and PG-2 were conducted on 100 million and 30 million MCMC chains, with sampling period every 10 000 and 3000 chains, respectively. For PG-1, the best-fit model was GTR + Γ4 in combination with a random local clock and Bayesian Skyline demographic model. On the other hand, the combination of a TVM + Γ4 substitution model, strict molecular clock, and constant population size was best-fitting for PG-2.

### Accessory genome profiling

For each isolate, the paired-end sequencing reads were trimmed using Trimmomatic (v0.38) [31], and input into SPAdes (v3.12.0; k-mer sizes of 21, 33, and 55 bp; error correction option) [32] to produce a *de novo* assembly. Only contigs of more than 500 bp were included in subsequent analyses. For each isolate, antimicrobial resistance (AMR) genes were detected directly from short reads using ARIBA (v2.12.0) [33], based on the curated ResFinder database [34], with a minimum alignment length of 50% and minimum nucleotide identity of 95% to consider a hit. The output results were then manually curated for confident hits.

Pan-genome profiles were constructed by Roary (v3.12.0) [35] for all 96 sequenced Sf6, using the default 95% BLASTP identity. The inputs for this analysis were assembled contigs annotated by Prokka (v1.13) [36]. To identify accessory genomes encompassing detected genes and their associated contigs, the assembly of each isolate was ordered against the chromosome sequence of Sb227 and the virulence plasmid pCP301 of *S. flexneri* 2a (accession number: NC_004851.1) using ABACAS (v1.3.1) [37]. The pCP301 was used instead of the virulence plasmid pSB4_227 of *S. boydii* 4, since the latter lacks several virulence determinants such as *mxi-spa*, *icsA*/*virG* and *virA* [38, 39]. The remaining contigs were then sequentially ordered against a set of plasmids and the *Shigella* resistance locus pathogenicity island of *S. flexneri* 2a (SRL-PAI, accession number: AF326777.3). The list of plasmids used in the aforementioned procedure is provided in Table 1. The plasmids were selected based on the results of plasmid typing analysis, which was performed using ARIBA on the highly curated PlasmidFinder database [33, 40]. blastn [41] was utilized to compare ordered contigs with the reference sequences. Artemis and Artemis Comparison Tool (ACT) [42] were used to visually inspect the presence of specific genetic elements in the isolates.

**Table 1.** Reference plasmids used for investigation of the accessory genome of *Shigella flexneri* 6. The table lists all reference plasmids used for ordering the assembled contigs of 96 *S *. *flexneri* 6 isolates in Asia, in the same order of usage (see Methods)

Order	Plasmid name	Original species	Accession no.
1	pCP301 (virulence plasmid)	* S. flexneri* 2a strain 301	NC_004851.1
2	pSS046_spA	* S. sonnei* strain Ss046	CP000641.1
3	pNUC	*S*. Typhimurium	KU852461.1
4	pRC960-1	* S. flexneri* Y strain RC960	KY848295.1
5	pLF82	* E. coli* strain LF82	CU638872.1
6	pCTXM3_020032	* E. coli* strain WCHEC020032	CP034964.1
7	p3521	* E. coli *	GU256641.1
8	pBS512_33	* S. boydii* CDC 3083–94	CP001059.1
9	RCS87_p	* E. coli* strain ECOR 18	LT985298.1

## Results

### The population structure and evolutionary history of *Shigella flexneri* 6 in Asia

Our study included 96 Sf6 isolates, which were collected in previous diarrhoeal surveillance studies in Vietnam (1995–2010) [43, 44], Thailand (1987–2005) [45], Cambodia (2005–2007), and Bhutan (2011–2013) [46]. *In silico* MLST, as implemented in ARIBA using the *Escherichia coli *’s seven housekeeping genes (*adk*, *fumC*, *gyrB*, *icd*, *mdh*, *purA*, *recA*) confirmed that all Sf6 belonged to ST145, resembling previous findings [8]. Previous phylogenetic investigations on pan-* Shigella* species showed that Sf6 was phylogenetically clustered with *S. boydii* serotypes 2, 4, and 14 [9–11]. Read mapping using the *S. boydii* reference genome (Sb227) produced good mapping quality, with almost all unmapped regions pertaining to repetitive sequences.

**Fig. 1.**
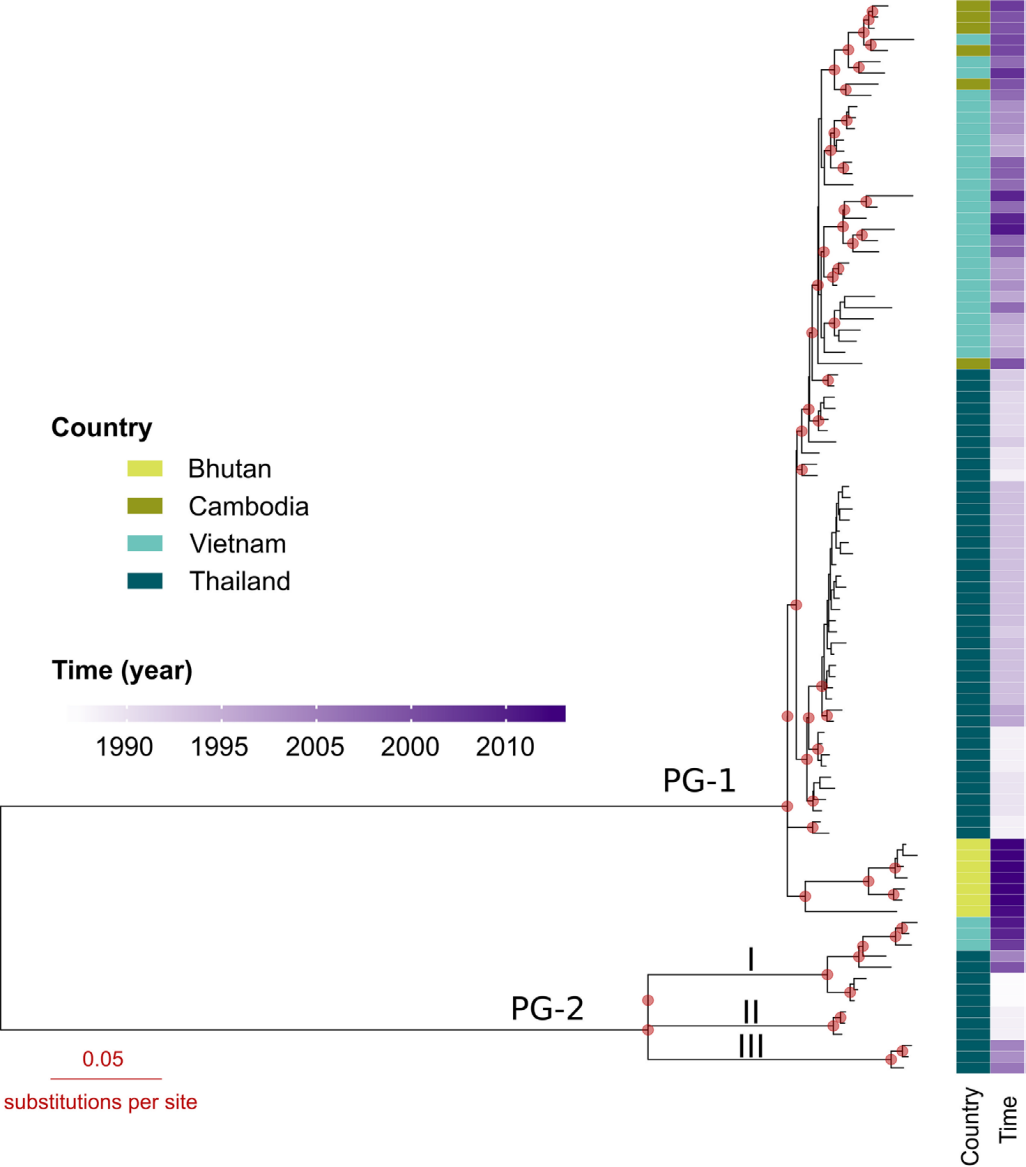
The phylogenetic structure of *Shigella flexneri* 6 in Asia. A maximum-likelihood phylogeny of 96 *S *. *flexneri* serotype 6 collected in four Asian countries (Thailand, *n*=53; Vietnam, *n*=30; Cambodia, *n*=6; and Bhutan, *n*=7). The phylogeny comprises two principal phylogenetic groups, which are herein referred as PG-1 (*n*=82) and PG-2 (*n*=14). PG-2 includes three subgroups: I, II, and III. The phylogeny is midpoint rooted. Red circles indicate nodes with bootstrap support ≥70. Illustrated by the columns on the right are the isolate’s country of origin and time of isolation (see key). The horizontal scale bar represents the number of nucleotide substitutions per site.

The constructed maximum-likelihood phylogeny delineated our 96 Sf6 into two phylogenetic groups (PG-1 and −2), both of which were supported by bootstrap values of 100 (Fig. 1). The evolutionary distance separating the two PGs was estimated to be ~2442 SNPs. PG-1 included 82 isolates from the four sampled countries while PG-2 only contained 14 isolates originating from Thailand and Vietnam. As depicted in Fig. 1, PG-2 appeared to harbour greater genetic diversity, with isolates spanning a broad timeframe (1987–2010), and was comprised of three subgroups: I, II, and III. The distances from subgroup III to subgroups I and II were estimated to be 719 and 730 SNPs, respectively. Subgroups I and II, on the other hand, were separated from each other by 621 SNPs. Geographical clustering was notable for isolates belonging to PG-1 (Fig. 1). Specifically, isolates from Southeast Asian nations and Bhutan belonged to two separate subgroups. Within the Southeast Asia subgroup, the Vietnamese Sf6 were interspersed with those of neighbouring Cambodia, and were separated from the paraphyletic Thai isolates (Fig. 1). Further inspection showed that several Thai strains (*n*=9) were basal to the Vietnamese and Cambodian counterparts. These observations may reflect the local establishment of Sf6 in Asia and their cross-border propagation between neighbouring countries.

**Fig. 2.**
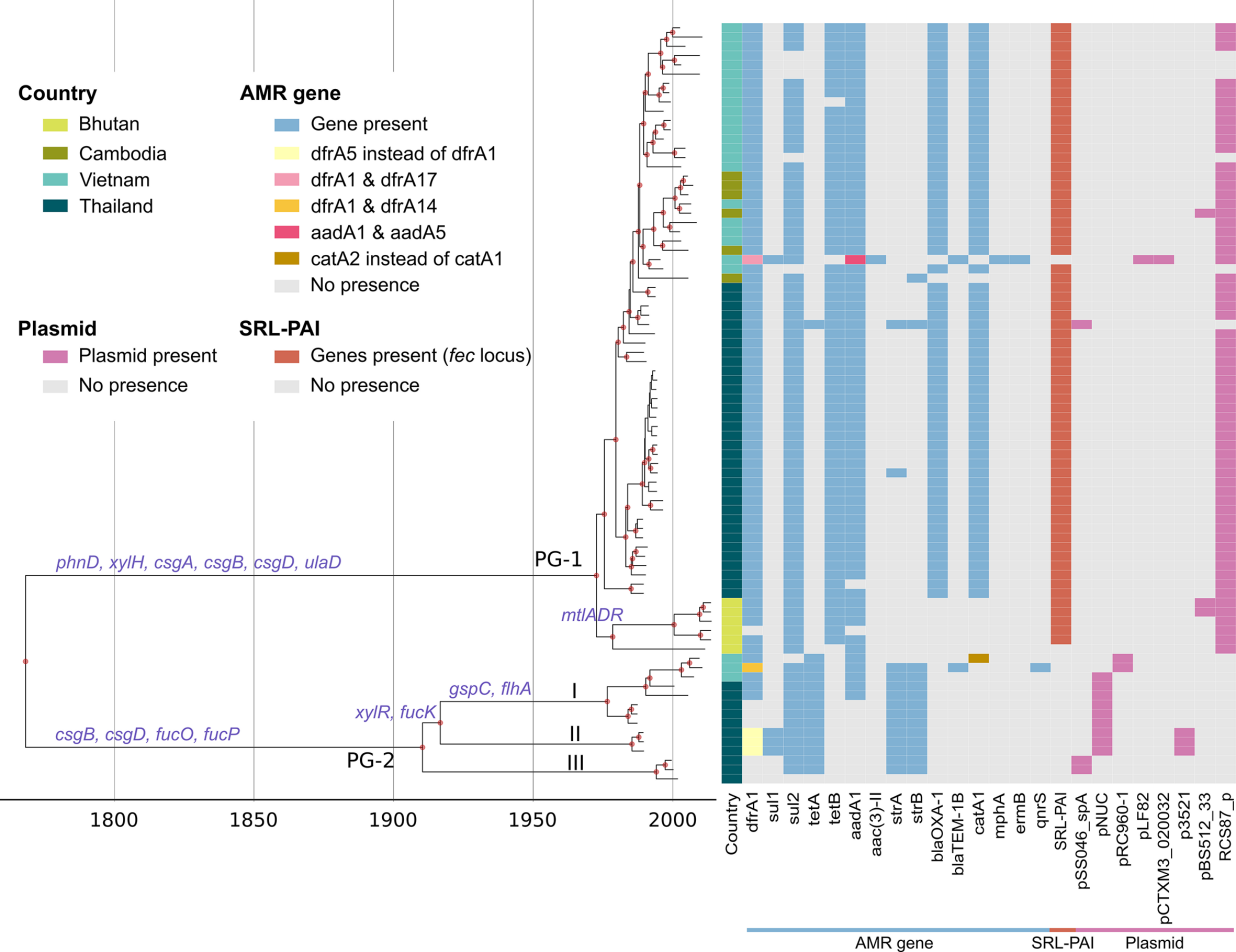
The temporal phylogenetic reconstruction of *Shigella flexneri* serotype 6 in Asia. The figure shows a maximum clade credibility phylogenetic reconstruction of 82 *S *. *flexneri* serotype 6 in four Asian countries (Thailand, *n*=45; Vietnam, *n*=25; Cambodia, *n*=6; and Bhutan, *n*=6). The phylogeny comprises two principal phylogenetic groups, which are herein referred as the major group (PG-1, *n*=68) and the minor group (PG-2, *n*=14). PG-2 includes three subgroups: I, II, and III. Red circles indicate posterior probability support ≥90 % on internal nodes. Right-hand columns correspond to the countries of origin, the presence of several antimicrobial resistance (AMR) genes (blue), the presence of the *Shigella* resistance locus pathogenicity island (SRL-PAI) (brick-red), and the presence of different plasmid backbones (pink), respectively (see key). Gene inactivation and gene loss events (purple) are overlaid on their corresponding branches.

In order to estimate the time of divergence for the Asian Sf6, we utilized Bayesian phylogenetic inference (BEAST v1.8.3) [27] on a subset of 82 representative strains (with attached sampling date) selected across the entire phylogeny. The mean nucleotide substitution rate was calculated to be 1.04×10^−6^ substitutions per site per year (95% highest posterior density (HPD): 8.84×10^−7^ to 1.21×10^−6^). This rate is consistent with previous estimates from global evolutionary history analyses for *S. flexneri* (6.46–9.54×10^−7^, species-wide, excluding Sf6 [12]), *S. dysenteriae* serotype 1 (8.70×10^−7^ [47]), and *S. sonnei* (6.0×10^−7^ [48]). Our Bayesian analyses also estimated that the examined Sf6 population likely arose in the 18th century (95% HPD: 1715.6–1814.6) (Fig. 2). The Bayesian phylogenetic topology matched that inferred from the maximum-likelihood method. With PG-1′s estimated most recent common ancestor (MRCA) dating back to ~1972 (95% HPD: 1967.0–1977.0), it was younger than PG-2, which was estimated to descend from an MRCA circa 1910 (95% HPD: 1901.3–1930.6) (Fig. 2). The calculated substitution rates of the two PGs were comparable (PG-1: mean 1.09×10^−6^ substitutions per site per year, 95% HPD: 7.92×10^−7^ to 1.21×10^−6^; PG-2: mean 9.88×10^−7^, 95% HPD: 8.14×10^−7^ to 1.17×10^−6^). For PG-2, all subgroups (I, II, and III) diverged during the late 20th century (95% HPD of the MRCA of subgroups I: 1971.7–1979.0; II: 1982.9–1986.0; and III: 1991.3–1995.2). Additionally, both PG-1 and PG-2 were independently assessed using similar BEAST analyses. The resulting tMRCAs and substitution rates for the two PGs were consistent with those inferred from the entire phylogeny, demonstrating the robustness of our analyses.

### Antimicrobial resistance profile in *Shigella flexneri* 6

In order to investigate the AMR profiles of Sf6, we used a genotyping approach, supported by the consistency between AMR genotyping and phenotyping results in *Shigella* as reported previously [49]. The resistome of Sf6 revealed a similar trend as observed in other *Shigella* species, in which resistance to first-generation antimicrobials for shigellosis treatment is commonplace [12, 47, 48, 50, 51]. In particular, genetic elements conferring resistance to sulphonamide (*sul1* or *sul2*, *n*=89/96), trimethoprim (*dfrA* variants, *n*=89/96), and tetracycline (*tetA* or *tetB*, *n*=92/96) were prevalent across the phylogeny (Fig. 2). However, the elements carrying these AMR genes were variable across and within the PGs. In PG-2, the multidrug resistance (MDR) element (carrying *sul2*, *tetA*, and *strAB*) was detected in all except two isolates (*n*=12/14), but this element was co-transferred in two different plasmid backbones. These include the spA-like plasmid (recovered previously in *S. sonnei* [38]) in subgroup III, and the IncQ1 pNUC-like plasmid in most isolates of subgroups I and II. On the other hand, a single acquisition of plasmid RCS87_p (~6 kb, carrying *sul2*) in the MRCA of PG-1 explains the widespread resistance to sulphonamide in this PG. This, coupled with the acquisition of *dfrA1* (carried on a class II integron) in most PG-1 isolates, subsequently rendered prevalent resistance to co-trimoxazole in the 1970s.

Another notable MDR element in our Sf6 collection was the *Shigella* resistance locus pathogenicity island (SRL-PAI) [52], which confers resistance to tetracycline (*tetB*), chloramphenicol (*catA1*), ampicillin (*bla_OXA-1_*), and aminoglycosides (*aadA1*). This SRL-PAI was likely introduced into the MRCA of PG-1, but not in PG-2, at least 50 years ago, and has been persistently maintained (Fig. 2). However, we observed the loss of the *catA1* and *bla_OXA-1_* in all Bhutanese and one Cambodian isolate, showing that the SRL was subject to modification. Resistance to macrolides or quinolones was identified in one Sf6 isolate each. Both of these were isolated in Vietnam, lacked the chromosomal SRL-PAI, carried *bla*_TEM-1B_, and harboured large multidrug resistance plasmids (~75–87.7 kb). Specifically, one isolate (PG-1) carried an IncFII plasmid (pCTXM3_020032-like) co-transferring five AMR genes (*mphA*, *ermB*, *sul1*, *aadA5*, and *dfrA17*), conferring additional resistance to macrolides. The other isolate (PG-2) harboured a pRC960-1-like plasmid co-transferring seven AMR genes (*tetA*, *sul2*, *strAB*, *dfrA14*, *bla*_TEM-1B_, and *qnrS),* predictively conferring additional resistance to quinolones. This isolate’s closest relative was also likely to have acquired a similar plasmid, but the MDR region (with the exception of *tetA*) had been lost (Fig. 2).

### Virulence determinants

Pan-genome analysis indicated the presence of 5894 genes in 96 examined Sf6, which consisted of 3468 core genes and 2426 accessory genes. Various virulence determinants were ubiquitous across the collection. These include the virulence plasmid and the aerobactin biosynthesis cluster (*iutA*, *iucABCD*), the deletions of which are known to attenuate the virulence of *S. flexneri* [53, 54]. The cell-entry region (*mxi-spa* type III secretion system) on the *Shigella* virulence plasmid is a prominent player in their pathogenesis [55]. In our Sf6 collection, the *mxi-spa* region was detected in 94/96 isolates. In each of these genomes, we detected the presence of a single large contig (33472–37893 bp) with 90 % nucleotide identity to the cell-entry region of *S. flexneri* 2a virulence plasmid pCP301. Likewise, the virulence genes *icsA/virG* and *virA* were identified in almost all isolates (*n*=94). Deletions of the cell-entry region, *icsA/virG*, and *virA* were scattered across the two principle PGs, and appeared to occur stochastically, likely as a result of culturing and/or storage conditions of the isolates. This was seen previously for the cell-entry region in the virulence plasmid pSB4_227 of *S. boydii* Sb227 [38]. In comparison to pCP301, certain loci were consistently missing in the virulence plasmid of *S. flexneri* 6. These include *sepA* (serine protease autotransporter for tissue invasion [56]⁠), *phoN1* (periplasmic non-specific acid phosphatase), *stbAB* (type II partitioning system), *rfbU* (UDP-sugar hydrolase), and *ipgH* (sugar phosphate transport protein) [38, 57]. These are also absent from the virulence plasmid pSB4_227 (* S. boydii *) as well as pSS_046 (* S. sonnei *), showing that their presence is probably a distinguishing feature of the *S. flexneri* 2a virulence plasmid.

For chromosomally encoded virulence factors, the SHI-1 pathogenicity island of examined Sf6 was more similar to that of *S. boydii* serotype 4 (Sb227) than of the *S. flexneri* 2a, since only *sigA* was detected while the *pic*/*set1AB* region (of *S. flexneri* 2a) was absent. As aforementioned, the *iut*/*iuc* operon, which encodes an aerobactin system (for iron acquisition), was present in all isolates. This suggested the existence of the *Shigella* pathogenicity island SHI-3. Besides this operon, Sf6 also harboured many other iron-uptake systems and associative regulators, which are commonly found in *Shigella spp*. These include the *sit* locus (*sitABCD*), *feo* locus (*feoABC*), *fhu* locus (*fhuABCD*), and the regulators *fur*, *fnr*, and *arcAB*. We also detected the presence of the complete enterobactin biosynthesis operon (*entABCDEFS – fepABCDGE – fes*) in all Sf6 isolates. This siderophore production system has only been found in some *S. boydii* strains, and its function in *S. flexneri* is currently disputed [12, 54, 58]. In *S. flexneri *, the system has been reported to be rarely utilized, but global phylogenetic analyses suggest that it is ancestral in the species and has been retained in several lineages [12, 58]. Closer inspection on the virulence gene repertoire of Sf6 highlighted the disparity between PG-1 and PG-2. One notable difference was the SRL-PAI-mediated *fec* locus (*fecIRABCDE*) encoding for ferric-dicitrate uptake [52], which was only present in PG-1 (Fig. 2). This locus has been proposed to confer selective advantages to *Shigella* by broadening the availability of nutrient iron [12, 59, 60].

### Reductive evolution

Substantial gene inactivation (pseudogenization) and gene loss are hallmarks in the genomic evolution of the *Shigella spp*. as they evolved into human-restricted pathogens. In this study, we identified several such instances in our Sf6 collection. The pseudogenized genes were those involved in metabolic functions and biosynthesis of cellular appendages (Table 2). Moreover, most of these targets exhibited differentiation between the two principal PGs. For instance, the *mtlADR* operon encoding for d-mannitol catabolism was only absent in the Bhutanese isolates of PG-1 (*n*=4). Genes *phnD* and *ulaD*, involved in phosphanate transport and l-ascorbate catabolism, respectively, were each inactivated by a nonsense mutation in all PG-1 descendants. On the other hand, the same genetic operon could undergo differing pseudogenization mechanisms in PG-1 and PG-2, eventually leading to the same predicted phenotypic consequences. The operon *xylRHGFAB*, responsible for d-xylose metabolism, was disrupted in different manners among the two PGs. In all PG-1 isolates, *xylH* was inactivated by a frameshift mutation, whereas in PG-2, the operon activator *xylR* was truncated and *xylH* remained intact. These predicted that d-xylose utilization was dysfunctional in both PGs and served as an exemplar of pseudogenization-mediated convergent evolution in Sf6.

**Table 2.** Reductive evolution at genes for metabolism and biosynthesis of cellular appendages. The presence/absence and intactness/disruption of genes involved in metabolic pathways and cellular appendages synthesis in the Sb227 genome (* S. boydii* 4) and in two principal phylogenetic groups of *S. flexneri* 6 in Asia (PG-1 and −2) are described. Asterisks annotate that the whole operon for d-xylose utilization was absent in one Thailand isolate within PG-1

Product / Biochemical reaction	Gene	Functions	Sb227 (CP000036.1)	* S. flexneri* 6	
				Major group (PG-1)	Minor group (PG-2)
**Metabolic function**					
d-mannitol	*mtlADR*	d-mannitol permease and catabolism	Intact	Whole operon loss in Bhutanese isolates (*n*=4)	Intact
Phosphonate	*phnD*	Phosphanate transport	Intact	Stop codon in all PG-1	Intact
l-ascorbate	*ulaD*	Anaerobic l-ascorbate degradation	Intact	Stop codon in all PG-1	Intact
d-xylose	*xylH*	d-xylose ABC transporter	Intact	Frameshift in all PG-1*	Intact
	*xylR*	d-xylose degradation regulator	Intact	Intact*	Truncated in subgroups I and II
l-fucose	*fucO*	L-1,2-propanediol oxidoreductase	Frameshift	Frameshift	Additionally truncated
	*fucP*	l-fucose/proton symporter	Truncated	Truncated	Gene loss in subgroups I and II; additional deletion in subgroup III
	*fucK*	l-fuculokinase	Stop codon	Intact	Frameshift in subgroups I and II
**Cellular appendages**					
Curli	*csgA*	Major curlin subunit	Intact	Gene loss in all PG-1	Intact
	*csgB*	Minor curlin subunit	Truncated	Gene loss in all PG-1	Additional truncation
	*csgD*	csgBAC transcriptional regulator	Intact	Gene loss in all PG-1	Disrupted by IS element
Flagella	*flhA*	Flagella biosynthesis	Intact	Truncated in one Bhutanese isolate	Truncated in subgroup I

We found that several operons, which had undergone some degrees of pseudogenization in the ancestral Sb227, continued to be disrupted in Sf6. For example, the operons encoding for curli structure (*csgBAC*), assembly, and secretion (*csgGFED*) were subjected to degradation in the Sb227 genome [61, 62], leaving only *csgA* and *csgD* intact. Detailed genetic characterization revealed that all PG-2 genomes harboured additional disruptive mutations in *csgB* and *csgD*, as compared to the Sb227 genome, while PG-1 likely lost nearly all genes belonging to these two operons. Likewise, several genes involved in l-fucose catabolism, including *fucO*, *fucP*, and *fucK*, were further disrupted or lost in PG-2. These genes were inactivated in the Sb227 genome, and genome-based metabolic models predicted that most *Shigella* species could not utilize l-fucose [63]. Therefore, the separate pseudogenization events in the *fuc* operon signify an ongoing evolutionary pathway towards exclusion of l-fucose metabolism in *Shigella *.

## Discussion

By constructing the phylogeny of Sf6 isolated from four Asian countries at whole genome level, we have identified two principal phylogenetic groups (PG-1 and −2) circulating in the region. The phylogenetic distance between the two PGs was considerable, and the MRCA of extant Asian Sf6 dated back to the 18th century. The two PGs were uneven in both size and tMRCA, with PG-1 being more frequently detected and emerging more recently than PG-2. The most distinguishing difference in their genetic repertoire was the exclusive presence of SRL-PAI in PG-1, which encodes multiple AMR genes and an iron uptake *fec* locus [12, 52, 60]. This island has been acquired independently and stably maintained on several occasions across multiple lineages of other *S. flexneri* serotypes [12] and of *S. dysenteriae* type 1 [47]. In the latter, the SRL-PAI was also the major genetic element associated with an MDR phenotype, and it was most prevalent in the recently emerging lineage [47]. Therefore, the SRL-PAI may grant a competitive advantage for PG-1, thus leading to its dominance in our collection. While our isolates mostly originated from diarrhoeal surveillance studies and should reflect the epidemiological trend of Sf6, the uneven distribution of samples across the two PGs might also be attributed to sampling bias in our data. The absence of Cambodian and Bhutanese isolates in PG-2 could be due to the narrower sampling windows in these two countries compared to those of Thailand (1987–2005) and Vietnam (1995–2010). Sf6 isolated in 1994 in Thailand constituted the largest proportion of our Thai isolates (*n*=19/53), and all these 1994 isolates belonged to PG-1. This spike reflected the occurrence of an Sf6 outbreak in 1994 in Thailand, which has been captured by a previous shigellosis surveillance study in the country (1993–2006) [64].

The major lineage PG-1, as well as all subgroups (I, II, III) of PG-2, were estimated to have emerged in the 1970s, with PG-1 undergoing clonal expansion in both Bhutan and Southeast Asia. However, the absence of non-Asian Sf6 genomes in our analysis did not allow us to conclude whether the two PG’s MRCAs emerged within or were introduced into Asia. Our previous study on other *S. flexneri* serotypes and *S. sonnei* in Southeast Asia has demonstrated that the extant progenies of these *Shigella* species likely got introduced into the region in the same time frame (1970s-90s) [13]. These findings together point to the intensity of multiple *Shigella* introduction events into Asia post-1970s, and subsequent propagation may have been facilitated by expanding population size and heightened human migration in Asia. Most of the examined Sf6 harboured several AMR genes, mostly conferring resistance to first-generation antimicrobials used to treat shigellosis (sulphonamide, trimethoprim, tetracycline, and chloramphenicol). On the other hand, resistance to more recent first-line antimicrobials (quinolone and macrolides) was rare (2/96 isolates). This finding mirrors the resistomes inferred from other *S. flexneri* serotypes in the same period in Southeast Asia, but is different from that of *S. sonnei *. More specifically, for other *S. flexneri* serotypes, the emergence of macrolide resistance was sporadic and showed no fixation, while *S. sonnei* isolated post-2010s showed an increase in macrolide resistance [13]. Likewise, resistances to third-generation cephalosporins and quinolone were both more prevalent in *S. sonnei *, with higher occurrence of *bla*_CTX-M_ variants and *gyrA*/*parC* mutations [13, 44, 51].

Detailed genetic investigation allowed us to catalogue a number of gene inactivation events in Sf6, and we focused on genes involved in metabolism and biosynthesis of cellular appendages (flagella, fimbriae, curli, etc.). Genes involved in the utilization of d-mannitol, d-xylose, phosphanate and l-ascorbate, which are intact in *S. boydii* 4 genome Sb227, were found to be inactivated in our examined Sf6 genomes. Components involved in these metabolisms have also been found to be pseudogenized or lost in other *Shigella* species (d-mannitol: lost in Sf301 (* S. flexneri* 2a) and pseudonized in Ss046 (* S. sonnei *); d-xylose: pseudonized and/or lost in Sd197 (* S. dysenteriae* 1), Sf301, and Ss046; phosphonate: pseudonized in Sd197 and lost in Sf301; l-ascorbate: pseudonized in Sd197 and Ss046) [38]. Additionally, evidence of convergent evolution between the two PGs was observed in differing pseudogenizations in d-xylose metabolism. Together, these results underscored the prominent role of reductive evolution affecting the metabolic flexibility of Sf6 and *Shigella spp*., which has been a hallmark of the pathogen’s adaptation for intracellular lifestyle [39, 65]. An exemplar for this is the contrast in l-fucose catabolism between *Shigella* and other extracellular enteric pathogens. Genes encoding l-fucose utilization have been inactivated in multiple *Shigella* species [38], and we also observed disruptions in such elements in our Sf6 collection. Similarly, genome-wide metabolic reconstruction indicated that most *Shigella* strains (7/8 tested, except *S. boydii* CDC 3083) could not sustain growth on l-fucose [63]. In contrast, other extracellular enteric pathogens (including *Campylobacter jejuni* [66], enterohemorrhagic *Escherichia coli* [67], *Salmonella enterica* serovar Typhimurium, and *Clostridium difficile* [68]) have been found to rely on l-fucose for their survival and pathogenesis, as it is abundant (incorporated in mucin) in the intestinal milieu or could be foraged from the host microbiota. Shedding of cellular appendages has been proposed as pathoadaptive in *Shigella spp*., differentiating them from many other Enterobacteriaceae [62]. Although curli are important in biofilm formation and host cell adhesion and invasion of *E. coli* and *Salmonella spp* [61, 62], the operon responsible for curli synthesis *(csgA-G*) has been independently inactivated across all *Shigella* species [38, 61]. In *Sf6*, it was noted that the operon carried additional reductions in PG-2 and many further gene losses in PG-1. These demonstrated the momentum of gene degradation in Sf6 when the bioprocess is not functional, possibly in order to reduce the energy expenditure on superfluous genetic elements that might have conferred a fitness cost to the bacteria [69].

Our research sheds light into the evolutionary history and genomic evolution of the understudied Sf6 in four Asian countries. Our findings, however, were constrained by certain limitations. Firstly, due to limited data, global isolates from other continents were not incorporated for phylogenetic context. Thus, our findings could not be extended to Sf6 circulating in other endemic regions such as Africa or South America. In addition, our samples were unevenly distributed among countries and years, and were over-represented by urban paediatric populations. These may bias interpretations, such as inducing a false impression of the country of origin, when there is much more data collected from a single country (Thailand in our case). Our investigations on the presence/absence and intactness/disruption of genetic elements were not exhaustive. Therefore, other features of genomic evolution events still remain unexplored.

Notwithstanding these limitations, our study builds a framework for future investigations into Sf6, which is the fourth most common *Shigella* serotype and a target for pan-* Shigella* vaccine development. Insights into Sf6 will help portray a more thorough representation of *Shigella* epidemiology and evolution, as well as informing the optimal development of therapeutic and public health interventions.

## Supplementary Data

Supplementary material 1
